# Supplementary material for: MetaRibo-Seq measures translation in microbiomes
Source: Nat Commun. 2020 Jun 29;11:3268. doi: 10.1038/s41467-020-17081-z (PMC7324362; doi:10.1038/s41467-020-17081-z)
Supplement: Supplementary file 10 — Supplementary Data 7 [file 41467_2020_17081_MOESM10_ESM.zip › File2/Confidence_High_Taxonomy/95373_out.krona.html]

Javascript must be enabled to view this page.

members
magnitude
magnitudeUnassigned
count
unassigned
taxon
rank

95373\_out

5

superkingdom
5
2

5
phylum
1239

class
5
186801

order
5
186802

186806
family
5

genus
5
1730


SRS056537\_contig\_number\_30804SRS063985\_contig\_number\_25799SRS143085\_contig\_number\_5113SRS148159\_contig\_number\_57685SRS148970\_contig\_number\_3583
5
species
39485
